# Plasmonic nanohole array for enhancing the SERS signal of a single layer of graphene in water

**DOI:** 10.1038/s41598-017-14369-x

**Published:** 2017-10-25

**Authors:** Amirreza Mahigir, Te-Wei Chang, Ashkan Behnam, Gang Logan Liu, Manas Ranjan Gartia, Georgios Veronis

**Affiliations:** 1 School of Electrical Engineering and Computer Sciences, Louisiana State University, Baton Rouge, Louisiana 70803 USA; 2 Center for Computation and Technology, Louisiana State University, Baton Rouge, Louisiana 70808 USA; 3 Intel Corporation, Ronler Acres Campus, Hillsboro, Oregon 97124 USA; 4 Department of Electrical and Computer Engineering, University of Illinois at Urbana Champaign, Urbana, Illinois 61801 USA; 5 Department of Mechanical and Industrial Engineering, Louisiana State University, Baton Rouge, Louisiana 70803 USA

## Abstract

We numerically design and experimentally test a SERS-active substrate for enhancing the SERS signal of a single layer of graphene (SLG) in water. The SLG is placed on top of an array of silver-covered nanoholes in a polymer and is covered with water. Here we report a large enhancement of up to 2 × 10^5^ in the SERS signal of the SLG on the patterned plasmonic nanostructure for a 532 nm excitation laser wavelength. We provide a detailed study of the light-graphene interactions by investigating the optical absorption in the SLG, the density of optical states at the location of the SLG, and the extraction efficiency of the SERS signal of the SLG. Our numerical calculations of both the excitation field and the emission rate enhancements support the experimental results. We find that the enhancement is due to the increase in the confinement of electromagnetic fields on the location of the SLG that results in enhanced light absorption in the graphene at the excitation wavelength. We also find that water droplets increase the density of optical radiative states at the location of the SLG, leading to enhanced spontaneous emission rate of graphene at its Raman emission wavelengths.

## Introduction

Since its discovery in 1974 surface enhanced Raman spectroscopy (SERS)^1–6^ has been used as a promising tool for detection and characterization of chemicals^7–18^. The Raman signal of molecules is strongly enhanced when they are adsorbed on the surface of metallic nanostructures, which exhibit local surface plasmon resonances. This SERS effect originates in the giant enhancement of the local electromagnetic field at the pump laser wavelength^19–23^ and of the local density of optical states (LDOS) at the Raman emission wavelengths^24–26^ in hotspots, due to surface plasmon resonances of the metallic nanostructures to which the target molecules are adsorbed^14,27–34^. Owing to its ultra-high sensitivity, SERS has shown promise as a powerful tool for chemical identification with applications in biochemistry, food sciences, environmental studies and forensics^35^. In many of these applications, such as in detection of pollutants in sea-water and in measurement of blood glucose^17^, SERS is measured in water.

The two-dimensional (2D) nature of graphene with a single atomic layer of sp^2^-bonded carbon atoms, and the easiness of its integration with plasmonic nanostructures has made graphene a useful probe for investigating the optical response of such nanostructures^22,36–44^. Graphene itself has also been used as a planar SERS substrate for other target molecules^38,40,45–48^. In addition, using graphene is useful for overcoming some of the problems associated with conventional SERS substrates, such as the photochemical reaction of molecules in direct contact with metallic nanostructures, and the continuum spectral background originated from fluorescence^49^.

In this paper, we numerically design and experimentally test a SERS-active substrate for enhancing the SERS signal of a single layer of graphene (SLG) in water. The SLG is placed on top of an array of silver-covered nanoholes in a polymer and is covered with water. The SLG experiences an enhanced optical overlap with the local fields at the surface of the SERS substrate. Here we report a large enhancement of up to 2 × 10^5^ in the SERS signal of the SLG on the patterned plasmonic nanostructure for a 532 nm excitation laser wavelength, when the SLG is covered with water. We provide a detailed study of the light-graphene interactions by investigating the optical absorption in the SLG, the density of optical states at the location of the SLG, and the extraction efficiency of the SERS signal of the SLG. The origin of the additional enhancement when water is placed on the graphene monolayer is that water leads to a red shift in the surface plasmon resonance, so that the excitation wavelength approximately matches the resonance wavelength of the structure^50^. We find that water droplets increase the confinement of electromagnetic fields on the location of the SLG that results in enhanced light absorption in the graphene at the excitation wavelength. We also find that water droplets increase the density of optical radiative states at the location of the SLG, leading to enhanced spontaneous emission rate of graphene at its Raman emission wavelengths.

## Results

### Graphene-covered plasmonic nanohole array

**Figure 1:**
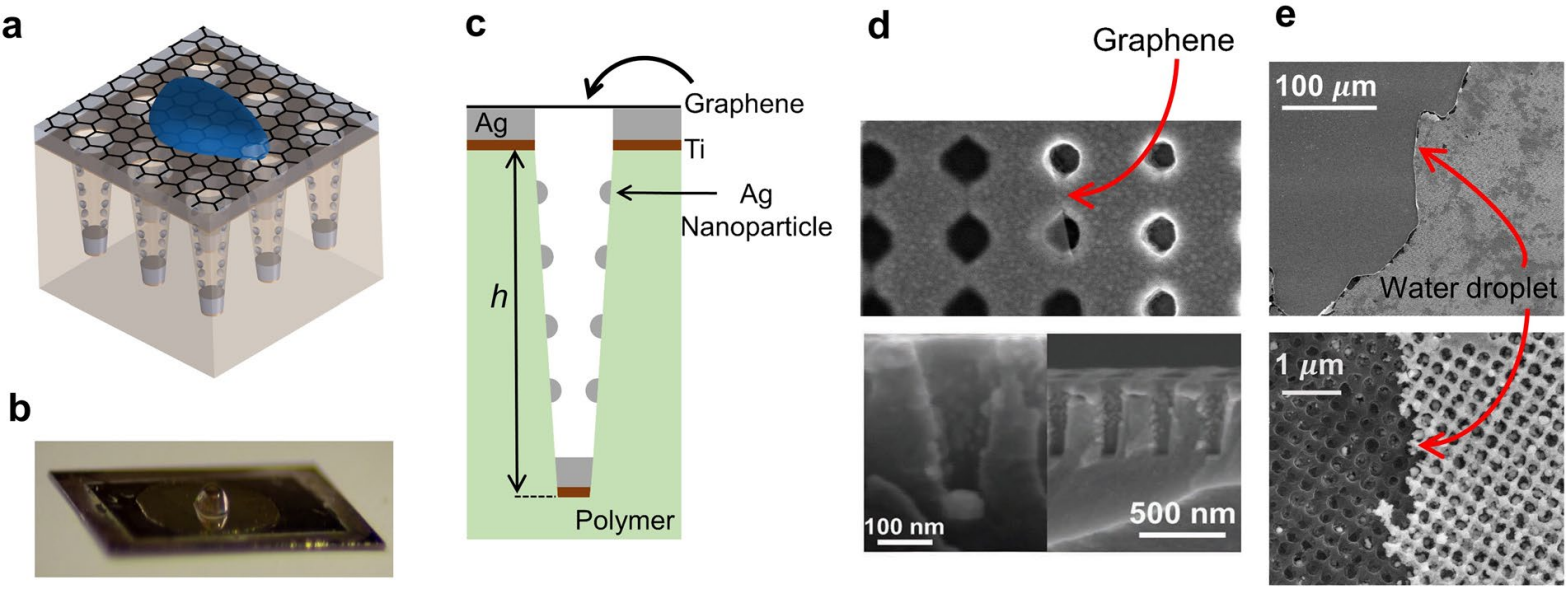
Schematic of plasmonic nanohole array covered by a single layer of graphene (SLG). (**a**) Nanohole array substrate with 350 nm lattice constant on polymer substrate (*n* = 1.56) supporting a SLG. A fraction of graphene surface is covered with water droplets. (**b**) Optical image of the sample shows that some regions on the surface of SLG are covered with water. (**c**) Cross-sectional view of one unit cell of the nanostructure. The thicknesses of silver and titanium layers are 90 nm and 9 nm, respectively. Nanoparticles are 40 nm in diameter. The cup depth *h* is 500 nm. Top and bottom hole diameters are 200 nm and 160 nm, respectively. (**d**) Top view (top) and cross-sectional view (bottom) SEM images of the nanohole array substrate. (**e**) SEM images of the nanostructure partially covered with water droplets.

We first design the SERS-active substrate consisting of a SLG placed on top of an array of silver-covered nanoholes in a polymer which is covered with water. A schematic of the designed system is shown in Fig. 1a. The electric field in the structure for a plane wave excitation is calculated using the finite-difference time-domain (FDTD) method. In order to maximize the enhancement of the SERS signal, we choose the periodicity of the array and the dimensions of the nanoholes so as to match the plasmon resonance of the structure with the excitation laser wavelength of 532 nm^39^. The periodicity of the square lattice of nanoholes and the cup depth *h* are chosen to be 350 nm and 500 nm, respectively (Fig. 1c). Top and bottom hole diameters are 200 nm and 160 nm, respectively.

**Figure 2:**
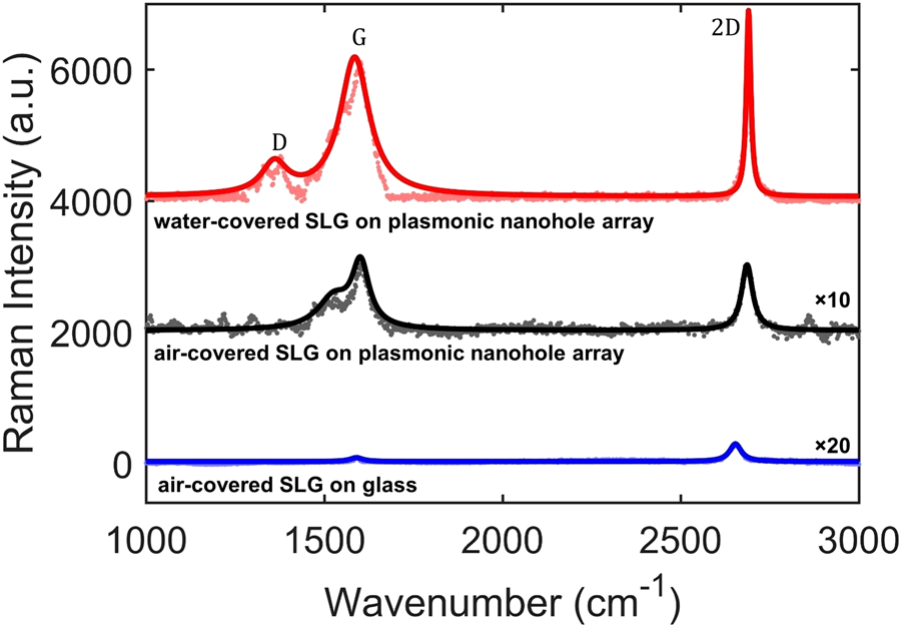
Observation of Raman response of the SLG. Raman spectra of SLG on different substrates, covered with either air or water. The spectra are shifted vertically for clarity. Incident laser power *P* at 532 nm is 211 *μ*W, and integration time *t* over which the spectrum was calculated is 10 s. Main peaks of Raman spectra of graphene are marked as D, G and 2D. The parameters of the plasmonic nanohole array are as in Fig. 1. The solid lines are Lorentzian fits to the data.

The chemical vapor deposition (CVD)-grown graphene layer was wet transferred onto the plasmonic nanohole array in which Titanium (Ti) and silver (Ag) layers are 9 nm and 90 nm in thickness, respectively (Fig. 1c, see Methods and Supplementary Information). The scanning electron microscope (SEM) image (Fig. 1d) and Raman spectra (Fig. 2) reveal the successful transfer of the graphene monolayer on top of the nanohole array. It can be clearly observed that the nanohole array exhibits high uniformity after the replication process (Fig. 1d,e). Moreover, due to the unique cup-shaped profile of the nanoholes, a dense array of 40 nm silver nanoparticles is formed on their sidewalls after metal deposition^51^. We found that, since the near field of the localized surface plasmons of these nanoparticles is negligible at the location of the graphene layer, the nanoparticles do not significantly contribute to the Raman enhancement. As we will see below, the proposed structure enhances the SLG Raman signal compared to an unpatterned structure. Surface plasmons at the interface of silver and the dielectric above it, and localized plasmons in the nanoholes promote strong light absorption in the SLG.

### Observation of SERS enhancement in SLG

Figure 1b,e show the optical and SEM images of the sample, respectively, with the SLG partially covered with water droplets. This sample enables us to consistently compare the SERS signal from the same SLG covered with either air or water on the same substrate. Figure 2 shows the measured SERS spectra of the SLG on the nanohole array covered with either air (black curve) or water (red curve). For comparison, the Raman signal of the SLG on glass is also shown (blue curve). We observe graphene Raman signal peak intensity enhancements of about 10-fold and 100-fold for the plasmonic nanohole array covered with air and water, respectively, compared to the graphene Raman signal for the glass substrate (covered with air). As discussed in the next section, this enhancement is due to the strong field confinement at the location of the SLG when it is covered with water at the pump laser wavelength, as well as the enhancement in the LDOS at the location of the SLG at its Raman emission wavelengths. It should be noted that, while the measured Raman signal spectra in conventional SERS-active substrates originate from an area comparable to the excitation wavelength, the enhancement of the Raman signal of the SLG predominantly comes from the plasmonic resonances that are extremely localized to areas of a few square nanometers on the surface of the sample^43^. If this difference in areas is taken into account, the enhancement in the SERS signal of graphene is calculated to be ~2 × 10^5^ for the plasmonic nanohole array covered with water (see Supplementary Information).

### Water-assisted SERS enhancement mechanism

In this sample the SERS signal of the SLG is enhanced through two physical processes of excitation rate enhancement and emission efficiency enhancement as follows^22,34,52–54^:

1 $$\gamma ={{\rm{\Gamma }}}_{{\rm{exc}}}{{\rm{\Gamma }}}_{{\rm{em}}}.$$

Here *γ* is the SERS signal enhancement. The excitation rate enhancement Γ_exc_ is defined as the enhancement in the light absorption in the SLG on the plasmonic nanohole array substrate covered with either air or water at the pump laser wavelength, compared with the absorption in the SLG on glass substrate, that is:

2 $${{\rm{\Gamma }}}_{{{\rm{exc}}}_{{\rm{a}},{\rm{w}}}}=\frac{{({P}_{{\rm{abs}}})}_{{\rm{a}},{\rm{w}}}}{{({P}_{{\rm{abs}}})}_{{\rm{g}}}},$$

where (*P*_abs_)_g_, (*P*_abs_)_a_ and (*P*_abs_)_w_ are the absorbed electromagnetic power in the SLG on glass substrate, in the SLG on plasmonic nanohole array substrate covered with air, and in the SLG on plasmonic nanohole array substrate covered with water, respectively. Here to elucidate the effect of water on the intensity of the SERS signal of the SLG when placed on the plasmonic nanohole substrate, we also investigate the optical response of the SLG when it is placed on the plasmonic nanohole array substrate and covered with air. The emission efficiency enhancement Γ_em_ is defined as the enhancement in the spontaneous emission rate of the SLG on plasmonic nanohole array substrate covered with either air or water at its Raman emission wavelengths, compared with the spontaneous emission rate of the SLG on glass substrate:

3 $${{\rm{\Gamma }}}_{{{\rm{em}}}_{{\rm{a}},{\rm{w}}}}=\frac{{({\gamma }_{{\rm{sp}}})}_{{\rm{a}},{\rm{w}}}}{{({\gamma }_{{\rm{sp}}})}_{{\rm{g}}}}.$$

Here (*γ*_sp_)_g_, (*γ*_sp_)_a_ and (*γ*_sp_)_w_ are the spontaneous emission rate of the SLG on glass substrate, on the plasmonic nanohole array substrate covered with air, and on the plasmonic nanohole array substrate covered with water droplets, respectively.

**Figure 3:**
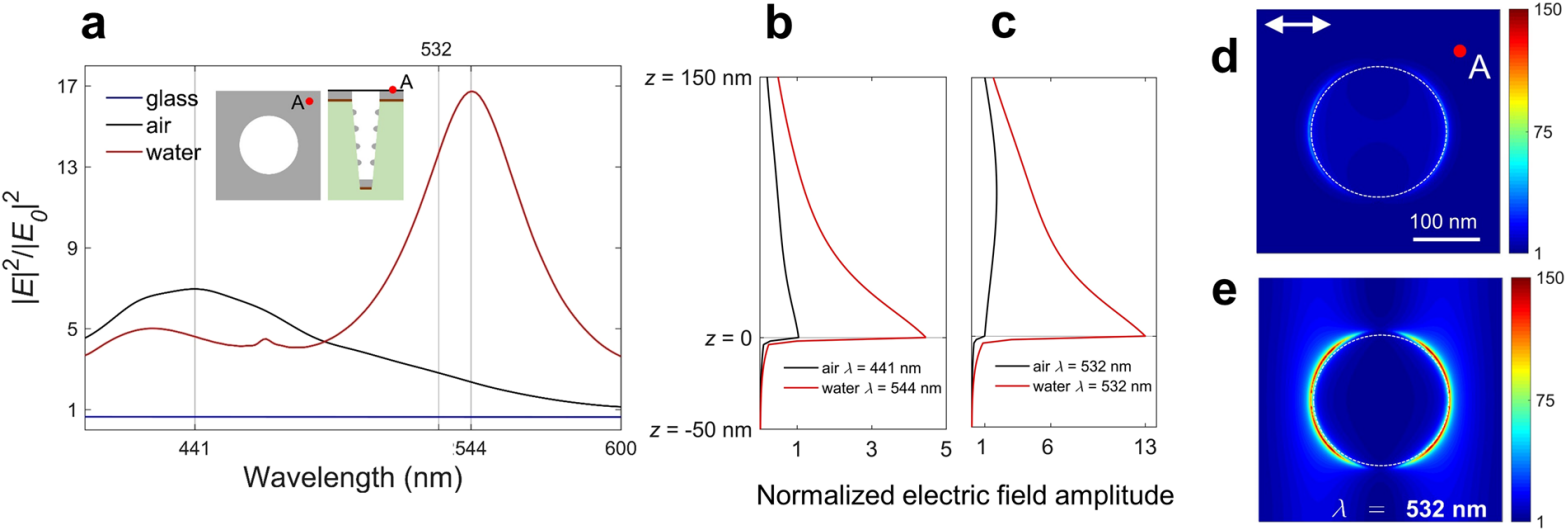
Average local electric field enhancement on the SLG surface. (**a**) Calculated average electric field intensity enhancement on the SLG surface on top of the nanohole array substrate as a function of wavelength for air (black) and water (red) on top of the SLG. Average electric field intensity enhancement on the SLG surface on glass substrate is also shown (blue). *E*_0_ is the field amplitude of the excitation source. The resonance wavelength is shifted from 441 nm to 544 nm after addition of water on the SLG. At the 532 nm pump laser wavelength the plasmonic nanohole array substrate enhances the spatially averaged electric field intensity on the surface of the SLG at *z* = 0 by a factor of ~5. The intensity of electric field is enhanced ~20 times on the surface of the plasmonic nanohole array substrate when it is covered with water droplets compared to the surface of a glass substrate. (**b**) Electric field profile across the silver-dielectric interface calculated at point A (Fig. 3d) at the resonant wavelengths of the structure covered with air (441 nm) and water (544 nm). In the simulation setup the SLG is placed at *z* = 0. Field amplitudes are normalized with respect to the field amplitude at the SLG location when it is covered with air. (**c**) Electric field profile across the silver-dielectric interface calculated at point A at excitation wavelength of 532 nm. Standing waves above the silver layer are expected since the pump laser excitation wavelength is away from the resonance wavelength of the structure with SLG on the nanohole array and covered with air. (**d**) Normalized electric field intensity with respect to the intensity of the excitation field on the surface of the SLG when graphene is covered with air. (**e**) Same as (**d**) except that graphene is covered with water. The polarization of the excitation source is also shown.

Hybrid graphene-metallic structures are beneficial in enhancing the local electric field dramatically, resulting in strong light absorption and Raman signal of the SLG. Absorption in the SLG is given by^55^:

4 $${P}_{{\rm{abs}}}(\omega )=\frac{1}{2}\,{\int }_{S}\,\sigma (\omega ){|E({\bf{r}},\omega )|}^{2}\,ds,$$

where *σ*(*ω*) is the surface conductivity of SLG, |*E*(**r**, *ω*)|^2^ is the intensity of the local electric field on the surface of the SLG, and *S* corresponds to the area of the SLG in one unit cell of the nanostructure. Absorption in the SLG is proportional to the local electric field intensity. To investigate changes in the absorption in the SLG due to addition of water droplets, we calculated the intensity of the local electric field on the surface of the nanohole array using full-wave 3D FDTD simulations. Figure 3a shows the field intensity averaged over the surface of the SLG under normal incidence when it is on a plasmonic nanohole array substrate and covered with air (black curve) or water (red curve) as a function of the wavelength. For comparison, the field intensity on the surface of SLG when it is placed on a glass substrate is also shown (blue curve). Placing water on the SLG changes the surface plasmon resonance condition of the metal-dielectric interface resulting in a red-shift in the resonance (441 nm→544 nm), which places it closer to the pump laser (532 nm). This results in increased light-graphene interaction and absorption in the SLG (see Supplementary Fig. S1). Addition of water droplets also increases the confinement of the local electric field in the vertical (*z*) direction at the interface between the nanohole array and the SLG (Fig. 3b,c). To quantify this confinement enhancement, the electric field intensity profile along the *z* direction is calculated at point A (as shown in the inset of Fig. 3a) across the interface of metal and air (black curve) or metal and water (red curve) at their respective plasmon resonances (441 nm for silver-air, and 544 nm for silver-water). Results are shown in Fig. 3b. It should be noted that the SLG is placed at *z* = 0 in the simulation setup (see Supplementary Information), and its effect on electric field intensity is negligible due to its small thickness. For ease in comparison, graphs are normalized to the electric field intensity at the silver-air interface. The silver-water field profile shows ~4.3 times enhancement on resonance in the local electric field at the surface of the SLG compared to the silver-air profile. This leads to stronger peak intensity of the averaged local field on the SLG covered with water at 544 nm compared to the peak intensity of the averaged local field on the SLG covered with air at 441 nm (Fig. 3a). Figure 3c shows the same electric field profiles at the excitation pump laser wavelength of 532 nm. The relative enhancement in the intensity of the electric field at the surface of the SLG is ~13 times, when water is added on top of the SLG, resulting in stronger optical overlap with the SLG, and thus enhanced absorption in it at the excitation pump laser wavelength. At the 532 nm pump laser wavelength the plasmonic nanohole array substrate covered with air enhances the spatially averaged electric field intensity at *z* = 0 (on the surface of the SLG) by a factor of ~5 compared to the glass substrate (Fig. 3a). From Eqs 2 and 4 we conclude that in this particular sample ${{\rm{\Gamma }}}_{{{\rm{exc}}}_{{\rm{a}}}}\approx 5$. Also from Fig. 3a and same equations we calculate ${{\rm{\Gamma }}}_{{{\rm{exc}}}_{{\rm{w}}}}\approx 20$. Figure 3d,e show the distribution of the intensity of electric field at 532 nm on the surface of the SLG on plasmonic nanohole array substrate covered with air and water, respectively. The excitation electric field is horizontally polarized. The cross-sectional view shows the enhancement in the intensity of the trapped electric field on the SLG at *z* = 0 after addition of water.

**Figure 4:**
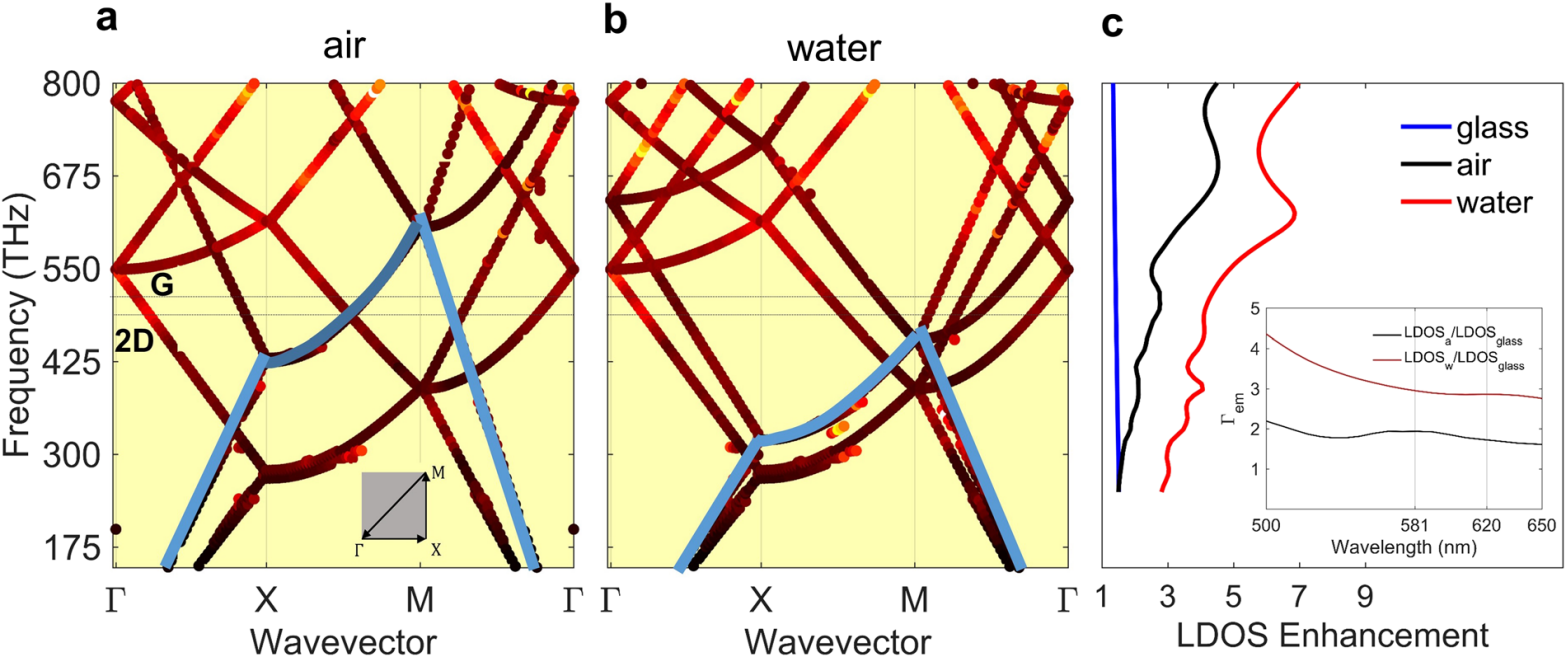
Calculated full band diagram and density of optical states of the nanostructure covered with air and water. (**a**) Band diagram of the nanostructure of Fig. 1a when the structure is covered with air. The blue curve shows the light line of air. The 2D Brillouin zone used in the calculation of the band diagram is shown in the inset. (**b**) Same as (**a**) except that the SLG is covered with water. The blue curve shows the light line of water. Placing water on the SLG pushes the light-line to lower frequencies, freeing up more radiative optical states at 581 nm (516 THz) and 620 nm (484 THz) for G and 2D Raman emission wavelengths of the SLG (shown with dotted lines). (**c**) Calculated averaged LDOS enhancement with respect to the DOS of a dipole emitter in free space when air (black) and water droplets (red) are placed on top of the graphene on the nanohole array. Average LDOS enhancement for a glass substrate is also shown (blue). The average was taken over the surface of the graphene in one unit cell of the nanohole array and also over all polarizations of the dipole emitter (See Supplementary Information). Placing water droplets on the SLG increases the LDOS at the Raman emission frequencies of the SLG. Inset shows the average enhancement in spontaneous emission rate. At the G and 2D Raman emission peaks of the SLG the averaged LDOS on the surface of the nanostructure covered with air and water, is increased ~2 and ~3 times, respectively, compared to the averaged LDOS on the surface of a glass substrate. Band diagram and density of states calculations are performed using 3D FDTD.

The intensified confinement in the electromagnetic field due to addition of water droplets results in increased LDOS at the surface of the SLG. To investigate the effect of water droplets on LDOS, we calculated the band diagram of the plasmonic nanohole array nanostructure covered with air and water, shown in Fig. 4a,b, respectively. Bands with energies below the light-line of air (solid blue curve in Fig. 4a) are trapped on the nanostructure, and are non-radiative^56,57^. The G and 2D Raman emissions of the SLG occur at the wavelengths of 581 nm (516 THz) and 620 nm (484 THz), respectively, for 532 nm excitation pump laser wavelength. When the SLG is on the plasmonic nanohole array covered with air, these emissions are mostly coupled to non-radiative states trapped in the nanostructure (Fig. 4a). These trapped modes cannot be detected in the far field. Varying the refractive index of the dielectric above the SLG, enables us to manipulate the bands in the diagram and turn the trapped modes to radiative modes (Fig. 4b). From Fig. 4b it can be seen that addition of water on the SLG shifts the light-line to lower frequencies, freeing more radiative states at the emission frequencies of G and 2D peaks, and resulting in enhancement in the Raman re-radiation of the SLG. This increase in the LDOS and radiative modes changes the spontaneous emission rate of an electric dipole located at the surface of the nanohole array as follows^26,58^:

5 $${\gamma }_{{\rm{s}}{\rm{p}}}=\frac{2\omega }{3\hslash {\varepsilon }_{0}}{|\mu |}^{2}\,{\int }_{s}\,\rho ({\bf{r}},\omega )\,ds,$$

where *μ* is the transition dipole moment of the emitter, *ε*_0_ is the dielectric permittivity of free space, *ħ* is the reduced Planck constant, and *ρ*(**r**, *ω*) is the LDOS. Based on equation (5), when the SLG is placed at the surface of the plasmonic nanohole array and is covered with water, it will experience enhancement in its spontaneous emission rate due to enhancement in the LDOS *ρ*(**r**, *ω*) which is given by^26,58^:

6 $$\rho ({\bf{r}},\omega )=\frac{2\omega }{\pi {c}^{2}}\{{\hat{{\bf{n}}}}_{{\rm{p}}}\cdot {\rm{Im}}\{{\rm{Tr}}[\overleftrightarrow{G}({\bf{r}},{\bf{r}})]\cdot {\hat{{\bf{n}}}}_{{\rm{p}}}\}\},$$

where *c* is the speed of light in free space, ${\hat{{\bf{n}}}}_{{\rm{p}}}$ is the orientation of the transition dipole of the emitter, and $\overleftrightarrow{G}$ is the dyadic Green’s function, which is the interaction of an emitter with the local electric field caused by its own radiation. Using Eq. 6, we calculated the averaged LDOS at the surface of the nanohole array. In the FDTD simulation, the position of an electric dipole emitter was varied in one unit cell of the nanostructure on a discrete 18 × 18 grid placed at 2 nm above the graphene for all three orientations of the dipole emitter (see Supplementary Information). The spatial average of the LDOS enhancement, with respect to the DOS of a dipole emitter in free space, over the surface of the nanohole array averaged over three polarizations of dipole emitter is calculated and shown in Fig. 4c. For comparison, the spatial average of LDOS enhancement, with respect to the DOS of a dipole emitter in free space, on the surface of the glass substrate is also shown (blue curve). The average LDOS is increased when water droplets are placed on the SLG. The inset of Fig. 4c shows the amount of increase in the averaged LDOS at *z* = 2 nm for nanostructure covered either air or water compared to the averaged LDOS on the surface of glass substrate in the wavelength range of interest (*λ* = 500 nm–650 nm). At 581 and 620 nm, corresponding to the G and 2D Raman emission peaks of the SLG, respectively, the averaged LDOS on the surface of the nanostructure is increased ~2 times compared to the averaged LDOS on the surface of a glass substrate. The averaged LDOS also increases ~3 times when the substrate is covered with water compared to a glass substrate. Thus, for this sample we conclude that ${{\rm{\Gamma }}}_{{{\rm{em}}}_{{\rm{a}}}}\approx 2$ and ${{\rm{\Gamma }}}_{{{\rm{em}}}_{{\rm{w}}}}\approx 3$. The total enhancement in Raman response of the SLG can then be calculated from equation (1) as *γ* ≈ 10 and *γ* ≈ 60 for the plasmonic nanohole array substrate covered with air and water, respectively, compared to the glass substrate.

**Figure 5:**
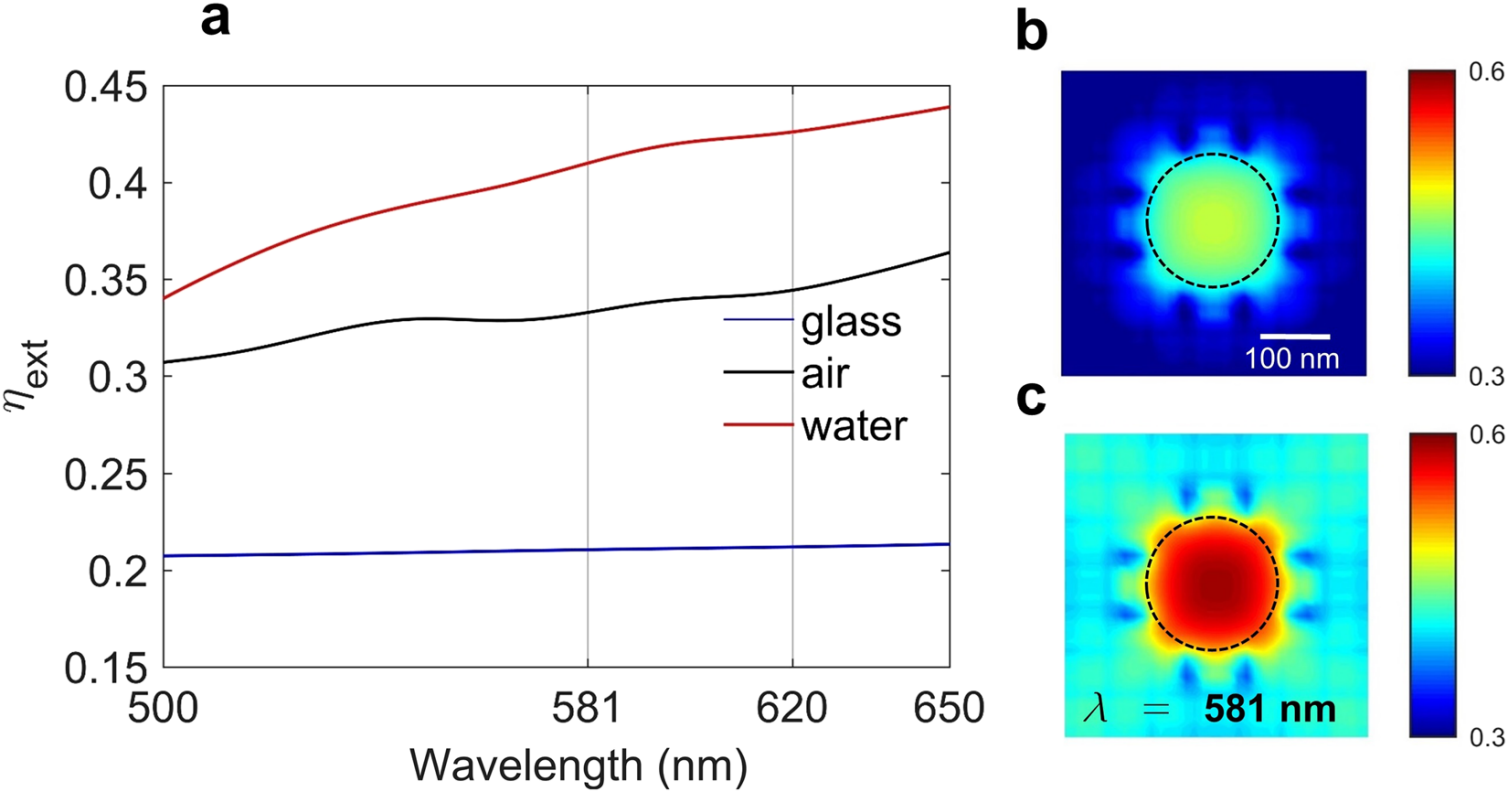
Extraction efficiency of emitters placed on the surface of the nanohole array. (**a**) Extraction efficiency of an electric dipole emitter placed on the surface of the nanohole array as a function of wavelength for air (black) and water (red). Extraction efficiency of a dipole emitter on a glass substrate is also shown (blue). The (*x*, *y*) position of the dipole emitter was varied to cover the entire surface of the SLG in one unit cell of the nanostructure and the extraction efficiency, defined as the ratio of radiated power along the +*z* direction to the total emitted power by the dipole, was calculated and averaged over the surface of the SLG and all three polarizations of the dipole emitter (See Supplementary Information). The extraction efficiency of a dipole emitter on the nanohole array covered with air and water is enhanced ~1.5 and ~2 times, respectively, compared to a dipole emitter on glass. (**b**) Extraction efficiency map of the nanostructure averaged over all three polarizations of the dipole emitter at 581 nm, corresponding to the G peak Raman emission wavelength of graphene, calculated at *z* = 2 nm, when the SLG is covered with air. (**c**) Same as (**b**) except that the SLG is covered with water.

A critical parameter in collecting the Raman re-radiation of a sample is the extraction efficiency, which is calculated as the ratio of the radiated power from an emitter which can be extracted at the detector, to the total emitted power by the emitter. Here we investigate the effect of addition of water on the sample on the extraction efficiency of the SERS signal of the SLG. In the experimental setup the detector is located above the sample (*z* > 0 in Fig. 3b). In simulations the extraction efficiency was therefore calculated as the ratio of the power radiated towards the +*z* direction to the total emitted power from an electric dipole emitter placed on the surface of the nanostructure. The location and polarization of the electric dipole emitter was varied as in the LDOS calculation (see Supplementary Information). Figure 5a shows the spatially averaged extraction efficiency as a function of wavelength averaged over all three orientations of the dipole emitter when the plasmonic nanohole array is covered with either air (black curve) or water (red curve). The averaged extraction efficiency is also calculated for a dipole emitter on glass substrate (blue curve). The average extraction efficiency increase is ~50% at the emission wavelengths of G (581 nm) and 2D (620 nm) peaks of the SLG on the air-covered plasmonic nanohole array substrate compared to the glass substrate. In addition, at the emission wavelengths of SLG, the average extraction efficiency increases 2 times for a dipole emitter on the plasmonic nanohole array substrate covered with water compared to a dipole emitter on the glass substrate (Fig. 5a). The spatial dependence of the extraction efficiency for the plasmonic nanohole array substrate covered with air and water is shown in Fig. 5b,c, respectively. If we include this enhancement in the extraction efficiency of the Raman signal of the SLG in our theoretical calculations by multiplying equation (1) by ${\eta }_{{{\rm{ext}}}_{{\rm{a}}}}\approx 1.5$, and ${\eta }_{{{\rm{ext}}}_{{\rm{w}}}}\approx 2$ we conclude that the total enhancement of the SERS signal of the SLG when it is on the plasmonic nanohole array substrate and covered with air is (*γ*_*tot*_)_a_ ≈ 15 when compared to the SLG on glass substrate. In addition, the total enhancement of the intensity of the SERS signal of the SLG when placed on the plasmonic nanohole array and covered with water is (*γ*_*tot*_)_w_ ≈ 120. The addition of water droplets not only traps light on the location of the SLG leading to enhancement of both absorption and density of optical states, but also increases the extraction efficiency of the SERS signal of graphene.

These numeric calculations are in good agreement with the experiments in which we measured (*γ*_*tot*_)_a_ ≈ 10 and (*γ*_*tot*_)_w_ ≈ 100. It should be noted that the area over which the Raman spectra is measured is comparable with the excitation wavelength. However, the Raman enhancement in the graphene is mostly attributed to the plasmonic resonances (also known as hotspots) on the surface of the nanohole array which are typically on the order of few nanometers^43^. If this difference in the excitation area and the size of hotspots on the sample are taken into account the actual enhancement factor achieved is up to 2 × 10^5^ (see Supplementary Information).

## Discussion

We numerically designed and experimentally tested a SERS substrate to enhance the Raman signal of a monolayer of graphene in water. We showed a large enhancement of Raman signal (of up to 2 × 10^5^) from graphene on a SERS substrate consisting of a plasmonic nanohole array and covered with water. The enhancement is due to the increase in the confinement of electromagnetic fields on the location of the SLG that results in enhanced light absorption in the graphene at the excitation wavelength. Water droplets also increase the density of optical radiative states at the location of the SLG, leading to enhanced spontaneous emission rate of graphene at its Raman emission wavelengths. We believe the proposed sample can be used in biomedical and environmental applications that require SERS measurements in water.

## Methods

### Graphene growth and transfer

Graphene layers were CVD-grown on both sides of a Cu substrate. A protective PMMA layer was deposited on one side and the unprotected graphene and Cu were etched by O_2_ plasma and FeCl_3_ solution, respectively. The remaining graphene/polymer-scaffold stack was wet transferred to the surface of the plasmonic nanostructure. Finally, the PMMA was removed in a dichloromethane and methanol solution (see Supplementary Information for more details). The measured 2D band of the graphene sample had a symmetric Lorentzian shape with a full width at half maximum of ~39 cm^−1^ which corresponds to a single layer of graphene. In addition, the measured drop in the transmission spectra when the graphene sample was placed on top of the glass substrate was ~2.3% which again corresponds to a single layer of graphene. Finally, the high 2D to G peak intensity ratio for the graphene sample measured on several different regions using a 633 nm laser also corresponds to a single layer of graphene.

### Characterizations and measurements

Renishaw PL/Raman micro-spectroscope system was used for Raman signal measurements. 532 nm Nd:YAG laser was used as the excitation light source. 50× long working distance objective lens was used to focus/collect incident light and Raman signal onto/from the surface of the graphene-metallic device. The range of measured wavenumbers was from 200 to 3000 cm^−1^.

### Numerical simulations

A commercial package (Lumerical FDTD Solutions) was used to simulate absorption, field enhancement, band diagram, LDOS and extraction efficiency. A fine mesh size of 1 nm was used throughout the simulation domain. The dielectric permittivity of silver was taken from CRC data^59^. Graphene was simulated as a 2D object based on its surface conductivity. The surface conductivity was tuned to give 2.3% light absorption in a single graphene layer suspended in air in the visible and NIR wavelength ranges^60^. In the absorption and field calculations periodic boundary conditions were used along the *x* and *y* directions, and perfectly matched layer (PML)^61^ was used along the *z* direction. The structure was excited by a broadband *x*-polarized plane wave. In band diagram calculations, Bloch boundary conditions were used along *x* and *y* ^61^. An electric dipole emitter was used for Green’s function calculations, from which the local density of optical states and spontaneous emission rate were derived. A large nanohole array substrate (9 × 9 unit cells) was used with PML boundary conditions along all boundaries for the Green’s function calculations (see Supplementary Information for more details).

## Electronic supplementary material

Supplementary Information
