# Supplementary material for: Plasmonic nanohole array for enhancing the SERS signal of a single layer of graphene in water
Source: Sci Rep. 2017 Oct 25;7:14044. doi: 10.1038/s41598-017-14369-x (PMC5656589; doi:10.1038/s41598-017-14369-x)
Supplement: Supplementary file 1 — Supplementary Information [file 41598_2017_14369_MOESM1_ESM.pdf]

Supplementary Information

Plasmonic nanohole array for enhancing the SERS signal of a single layer of graphene in water

Amirreza Mahigir[1,2], Te-Wei Chang[3], Ashkan Behnam[4], Gang Logan Liu[4], Manas Ranjan Gartia[5], and Georgios Veronis[1,2]

[1]School of Electrical Engineering and Computer Sciences, Louisiana State University, Baton Rouge, Louisiana 70803, USA.

[2]Center for Computation and Technology, Louisiana State University, Baton Rouge, Louisiana 70808, USA.

[3]Intel Corporation, Ronler Acres Campus, Hillsboro, Oregon 97124, USA.

[4]Department of Electrical and Computer Engineering, University of Illinois at Urbana Champaign, Urbana, Illinois 61801, USA.

[5]Department of Mechanical and Industrial Engineering, Louisiana State University, Baton Rouge, Louisiana 70803, USA.

1 Graphene growth

Graphene was grown on a Cu substrate (Alfa Aesar, 0.025 mm thick, 99.8%) treated in a dilute 2:1 de-ionized H_2O to hydrochloric acid (HCl) solution for 3 minutes. Growth was done in a 1-inch CVD furnace. First, the Cu foil was annealed for 1 hr at 1000 °C in order to increase Cu grain size under a H_2/Ar flow at ∼1 Torr. Next, methane was introduced for 20 to 25 min. Finally, the furnace was cooled down to room temperature under methane flow for ∼1 to 2 hrs. Graphene growth was confirmed on both surfaces (i.e. top and bottom) of the Cu foil with Raman spectroscopy.

2 Graphene transfer

First we deposit PMMA layers (495K A2 and 950K A4) with a final thickness of ∼300 nm to graphene/Cu/graphene. Each PMMA layer was spun at 3000 rpm for 30 s and baked at 200 °C for 2 min. The final thickness of PMMA scaffolds was determined by profilometry to be ∼ 290 nm±10 nm. Next, graphene is etched from the

unprotected polymer-free graphene/Cu/graphene surface by O_2 plasma ($\sim$80 W for 30 s). Then the Cu foil is etched overnight in a $FeCl_3$ solution. This process results in a graphene/polymer-scaffold stack floating on the surface of the etchant solution. Subsequently, this stack is transferred with a clean glass slide to three successive 15 min. baths (DI H_2O, 2:1 DI H_2O:HCl, and DI H_2O) to clean and remove residues from the Cu etching process. Finally, the graphene/polymer-scaffold stack is removed from the last DI H_2O bath using a plasmonic substrate.

3 PMMA lift-off

To successfully remove PMMA without damaging the graphene layer, water was first removed by drying it in air for 2-3 hrs followed by a soft bake on a hot plate (50 °C for 5 min). The PMMA was removed by using 1:1 dichloromethane and methanol solution for 40-60 min., followed by simple degreasing with methanol, isopropanol, and DI water.

4 Fabrication of nanohole array substrate

Nanoimprint lithography method was utilized to prepare the nanohole photonic structures. The master mold consisting of a two-dimensional square array of nanopillar structures with a lattice constant of $\sim$350 nm was made using laser interference lithography on glass substrate. The two-dimensional square array was transferred to a flexible and optically transparent polyethylene terephthalate (PET) film by replica molding process. The master mold was first cleaned and silanized (Repel-silane ES GE Healthcare, Sigma) for 30 min. followed by ethanol and DI water rinse. A 10 μL drop of UV-curable polymer (NOA-61) was evenly spread on the top of the nanopillar master and a supporting PET sheet was carefully put on top of the polymer. The master mold along with the polymer and PET sheet was then exposed to UV-light (105 mW cm^{-2}) for 60 sec. After curing, the complimentary nanohole structures were transferred onto the polymer, which was then peeled off carefully from the master mold to complete the transfer process. In order to make the device surface plasmon active, 90 nm of silver along with a 9 nm titanium adhesive layer were deposited using electron beam deposition (Temescal six pocket E-Beam Evaporator).

5 Calculation of field enhancement, field confinement, and absorption in graphene

Finite-difference time-domain (FDTD) simulations were performed by utilizing Lumerical FDTD Solutions. The refractive index of silver and titanium were obtained from CRC data [1], and the refractive index of polymer and water were chosen 1.56 and 1.33 respectively. The graphene layer was modeled as a 2D object and its conductivity was tuned in a way that a suspended in air graphene layer shows 2.3% absorption in the visibile and near-infrared range. The mesh size was chosen 1 nm throughout the simulation domain which was terminated to periodic boundary conditions in x and y, and to perfectly mathced layers (PML) in z direction. A plane-wave was used to excite the nanostructure as the pump signal (normally incident from top) in the wavelength range of 400 nm to 900 nm. The polarization of the excitation field was along the x direction (Fig. 3d). The local electric field in the location of the SLG was obtained using a plane frequency-domain field monitor which was placed on the surface of the SLG. The average electric field over the surface of the SLG in one unit-cell of the nanostructure was calculated for both air and water on top.

The field profile along the z direction was obtained using a single-frequency plane-wave excitation at the resonance wavelength of 441 nm (silver-air), 544 nm (silver-water) and pump laser of 532 nm (Figs. 3b and 3c, respectively) [2]. The field profile was obtained by utilizing a line frequency-domain field monitor (along the z direction). The location of this field monitor corresponds to point A in Fig. 3d.

Figure S1 shows the absorption spectra of the SLG when it is entirely covered with air (black) and water (red). Due to increase in confinement of the field in the location of the SLG (on the surface of the nanohole array substrate) and enhanced light-graphene interaction, the total absorption in the SLG increased 26% when it was covered with water droplets.

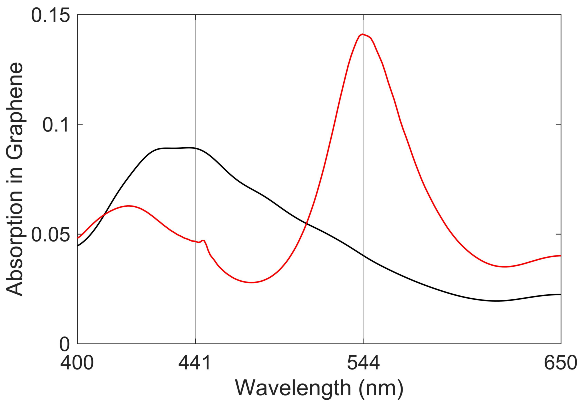

Figure S1: Absorption in the SLG as a function of wavelength when it is entirely covered with air (black) and water (red). Placing water droplets on the SLG increases the total absorption in graphene.

6 Calculation of the band structure

Through different simulations we calculated the band structure for the nanostructures covered with air and water. The Bloch boundary conditions were used along x and y and PML along z direction. A uniform mesh size of 5 nm was used for the entire simulation domain. A set of electric dipoles was used as point sources with random position and polarization to excite all optical modes. The k-vector was varied along the reduced 2D Brillouin zone (inset of Fig. 4a) of the plasmonic photonic crystal (nanohole array) and a set of time domain monitors with random positions recorded the electric field for different k-vectors. The spectra and resonances were obtained through Fourier transform of the recorded electric fields.

7 Calculation of local density of optical states and extraction efficiency

A nanostructure consisting of several number of periods of the structure was used to calculate the LDOS and extraction efficiency. PML boundary conditions were used along x, y and z. The mesh size was chosen 1 nm in the vicinity of the surface of the nanostructure and the SLG and 5 nm in other areas. Taking advantage of the symmetry of the structure, the position of an electric dipole emitter $\mathbf{r_0}$ was varied in one quarter of a unit cell of the nanostructure on a 18×18 grid (Figure S2) located 2 nm above the location of the SLG (SLG is located at $z = 0$) [3]. For each grid point and for each orientation of the dipole emitter (i.e. x, y and z) we calculated the Green's function, which is the electric field at $\mathbf{r}$ generated by a dipole emitter located at $\mathbf{r_0}$, and from it we obtained the LDOS. For instance, the z component of the Green's function

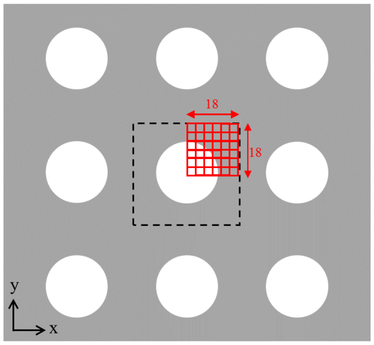

Figure S2: Schematic of the nanostructure used for calculation of LDOS and extraction efficiency. The position of the dipole emitter was varied on the 18×18 grid shown in red in the schematic.

was calculated as [4]:

$$G_{zz}(\mathbf{r}, \mathbf{r_0}; \omega_0) = \frac{E_z(\mathbf{r}, \mathbf{r_0}) c^2 \epsilon_0 \epsilon_r}{\mu \omega_0^2}, \tag{1}$$

where $G_{zz}(\mathbf{r}, \mathbf{r_0}; \omega_0)$ is the z component of the Green's function due to a dipole emitter oriented along z, $E_z(\mathbf{r}, \mathbf{r_0})$ is the z component of the electric field at $\mathbf{r}$ generated by the dipole emitter located at $\mathbf{r_0}$, and μ is the dipole moment of the emitter. The partial density of optical states along the z direction was calculated using [4]:

$$\rho_z(\mathbf{r_0}, \omega_0) = \frac{6\omega_0}{\pi c^2}\Big\{\text{Im}[G_{zz}(\mathbf{r_0}, \mathbf{r_0}, \omega_0)]\Big\}, \tag{2}$$

where $\rho_z(\mathbf{r_0}, \omega_0)$ corresponds to the partial density of optical states at a given location $\mathbf{r_0}$ and a given frequency ω_0. $G_{xx}(\mathbf{r_0}, \mathbf{r_0}, \omega_0)$ and $G_{yy}(\mathbf{r_0}, \mathbf{r_0}, \omega_0)$ were calculated with the same approach for the dipole emitters oriented along x and y, respectively, and from them $\rho_x(\mathbf{r_0}, \omega_0)$ and $\rho_y(\mathbf{r_0}, \omega_0)$ were obtained. The total density of optical states at $\mathbf{r_0}$ was calculated by averaging over all the orientations:

$$\rho(\mathbf{r_0}, \omega_0) = \frac{1}{3}\Big\{\frac{6\omega_0}{\pi c^2}\text{Im}[G_{xx}(\mathbf{r_0}, \mathbf{r_0}, \omega_0)] + \frac{6\omega_0}{\pi c^2}\text{Im}[G_{yy}(\mathbf{r_0}, \mathbf{r_0}, \omega_0)] + \frac{6\omega_0}{\pi c^2}\text{Im}[G_{zz}(\mathbf{r_0}, \mathbf{r_0}, \omega_0)]\Big\} = \frac{2\omega_0}{\pi c^2}\text{Im}\Big\{\text{Tr}[\overleftrightarrow{G}(\mathbf{r_0}, \mathbf{r_0}, \omega_0)]\Big\}. \tag{3}$$

The LDOS curves as a function of frequency are then obtained by taking the average of LDOS over all grid points for the two cases of the nanostructure entirely covered with air and water.

The extraction efficiency η was calculated as the ratio of the emitted power of a dipole emitter which radiates towards the $+z$ direction (P_z), and the total generated power by the dipole (P_t):

$$\eta = \frac{P_z}{P_t}. \tag{4}$$

In the FDTD simulations a plane frequency-domain power monitor was placed on top of a dipole emitter to record the emitted flux along the $+z$ direction. A closed box of frequency-domain power monitors was also used to calculate the total radiated power by the emitter. Using the same procedure described above for calculation of the LDOS, we varied the position of a dipole emitter on a 18×18 grid (Figure S2). For each grid point we calculated η in Eq. 4 for all orientations of the dipole emitter (i.e. x, y and z). Finally, by taking the average over all grid points for all orientations for the two cases of air and water on top of the SLG, we obtained the extraction efficiency as a function of wavelength (Fig. 5).

8 Raman enhancement factor

The area over which the Raman spectra are measured is comparable with the excitation wavelength. However, the Raman enhancement in the graphene is mostly attributed to the plasmonic resonances (also known as hotspots) on the surface of the nanohole array which are typically of the order of a few nanometers in diameter [5, 6, 7]. Here we take into account the difference between the excitation area and the size of the hotspots

on the sample. As suggested in [5], the graphene Raman enhancement factor EF for graphene-plasmonic structures can be defined as:

$$\mathrm{EF} = \frac{I(\mathrm{patt})/A(\mathrm{hotspot})}{I(\mathrm{unpatt})/A(\mathrm{excitation})}, \tag{5}$$

where in our structure $I(\mathrm{patt})$ and $I(\mathrm{unpatt})$ are the measured intensities of G and 2D peaks of Raman spectrum of graphene on water-covered nanohole array and glass substrate, respectively. Also, $A(\mathrm{hotspot})$ and $A(\mathrm{excitation})$ are the areas of hotspot and excitation spot on the sample, respectively. The SERS measurments show that in our sample $\frac{I(\mathrm{patt})}{I(\mathrm{unpatt})} \approx 100$. For 532 nm excitation wavelength with an objective of 50$\times$ and NA of 0.9 we measured the excitation area size as 1.1 μm. Also, based on the electric field intensity profile on the surface of the nanohole array in one unit cell of the nanosctructure, we can calculate the hotspot size. Here the hot spot size is approximated as the surface area of one of the crescents (Figure S3), for which $A(\mathrm{hotspot}) \approx 620$ nm^2. Therefore the total enhancement factor can be calculated as EF $=$ $100 \times \frac{1.1\mu\mathrm{m}\times 1.1\mu\mathrm{m}}{620\mathrm{nm}^2} \approx 2 \times 10^5$.

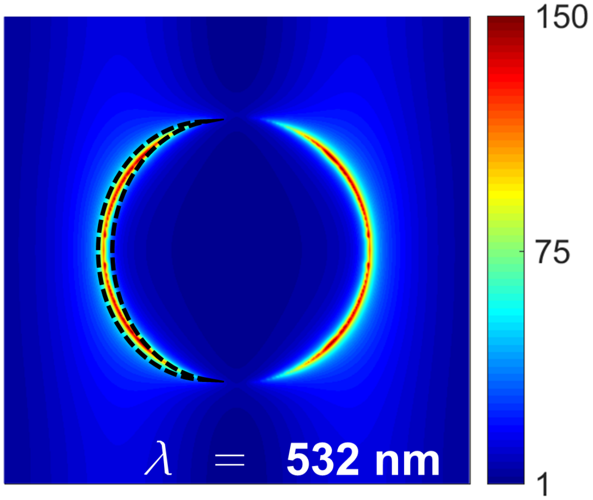

Figure S3: Elecric field intensity distribution on the surface of the graphene in one unit cell of the nanostructure. The sample is covered with water. The dashed black line encloses the surface area of the hotspot.

References

[1] Weast, R. C., Astle, M. J. & Beyer, W. H. *CRC Handbook of Chemistry and Physics* (CRC Press, 1988).

[2] Maier, S. A. *Plasmonics: Fundamentals and Applications* (Springer Science & Business Media, 2007).

[3] Xu, Y., Lee, R. K. & Yariv, A. Quantum analysis and the classical analysis of spontaneous emission in a microcavity. *Phys. Rev. A* **61,** 033807 (2000).

[4] Novotny, L. & Hecht, B. *Principles of Nano-optics* (Cambridge University Press, 2012).

[5] Wang, P. *et al.* Giant optical response from graphene-plasmonic system. *ACS Nano* **6**, 6244–6249 (2012).

[6] Linn, N. C., Sun, C. H., Arya, A., Jiang, P. & Jiang, B. Surface-enhanced Raman scattering on periodic metal nanotips with tunable sharpness. *Nanotechnology* **20**, 225303 (2009).

[7] De Angelis, F. *et al.* Breaking the diffusion limit with super-hydrophobic delivery of molecules to plasmonic nanofocusing sers structures. *Nat. Photonics* **5**, 683–688 (2011).
